# Un cas rare de luxation sous-talienne externe

**DOI:** 10.11604/pamj.2017.28.236.12320

**Published:** 2017-11-15

**Authors:** Said Zizah, Amine Marzouki, Kamal Lahrach, Fawzi Boutayeb

**Affiliations:** 1 Service de Chirurgie Orthopédique et Traumatologique A, Centre Hospitalier Universitaire Hassan II de Fès, Fès, Maroc

**Keywords:** Luxation externe, articulation sous-talienne, accident de sport, External dislocation, subtalar joint, sporting accident

## Abstract

La luxation sous-talienne est une lésion très rare. Il s'agit d'une urgence orthopédique. La variante externe est exceptionnelle. Elle se voit rarement après un accident de sport. Les auteurs rapportent le cas d'un footballeur qui a présenté une luxation sous talienne externe ouverte stade II de Couchoix Duparc suite à un accident de sport lors d'une compétition. Le traitement a été orthopédique. Après un an de recul, le résultat fonctionnel était satisfaisant.

## Abstract (en)

Subtalar dislocation is a very rare injury. It is an orthopedic emergency. External dislocation is exceptional. It uncommonly occurs after a sport accident. We here report the case of a footballer presenting with Cauchoix and Duparc type II open external subtalar dislocation following an accident during a sport competition. The patient underwent orthopedic treatment. At 1-year follow-up, functional outcome was satisfactory.

## Introduction

La luxation sous-talienne complète est une lésion rare, surtout lorsqu'elle est pure. Elle ne représente que 1% de l'ensemble des luxations observées en traumatologie [1]. La variante externe est exceptionnelle, elle représente 17% de ces luxations. Elle se définie comme une luxation simultanée des articulations sous-talienne et talo-naviculaire sans fracture de col du talus. Nous rapportons le cas d'une luxation sous talienne externe complète ouverte stade II de Couchoix Duparc survenue chez un sportif de haut niveau. Le traitement a été orthopédique avec un très bon résultat fonctionnel.

## Patient et observation

**Figure 1:**
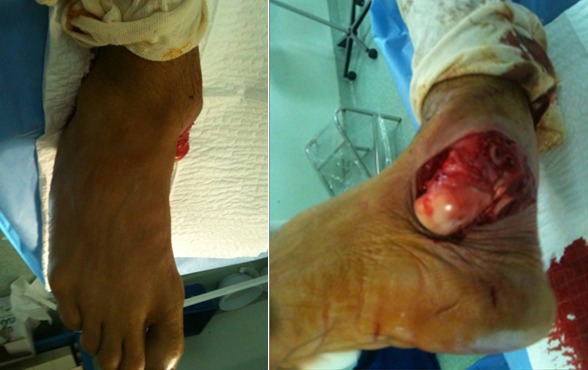
Aspect clinique d’une luxation sous-talienne externe gauche avec ouverture cutanée interne

**Figure 2:**
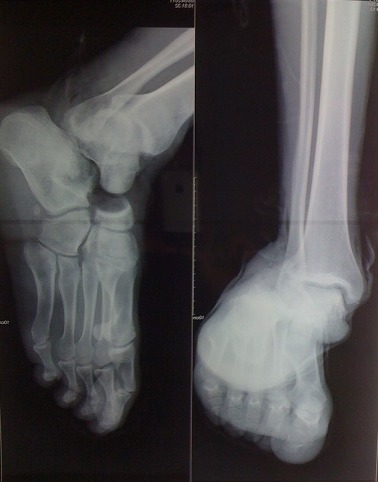
Radiographie standard de face et de profil de la cheville gauche montant une luxation sous-talienne externe

**Figure 3:**
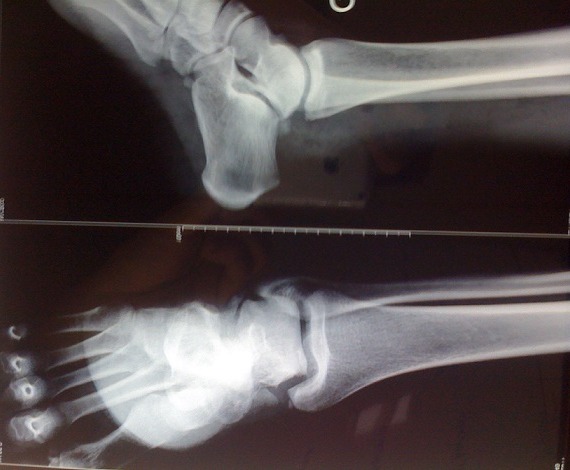
Aspect radiologique de la cheville gauche de face et de profil après réduction de la luxation sous-talienne externe

Il s'agissait d'un patient âgé de 29 ans, sportif de profession qui a été victime lors d'un match de football d'un traumatisme du pied droit à la suite d'un tacle avec pied bloqué contre le sol occasionnant chez lui des douleurs avec impotence fonctionnelle totale du membre inférieur droit. L'examen à l'admission trouvait une douleur localisée avec déformation de la région médiotarsienne et une ouverture cutanée stade II de Couchoix Duparc sur la face interne de la sous talienne (Figure 1). L'examen vasculo-nerveux était normal. Le bilan radiologique objectivait une luxation sous-talienne et talonaviculaire externe sans fracture associée (Figure 2). La réduction orthopédique a été réalisée en urgence, après parage de la plaie, sous anesthésie générale par la manœuvre d'arrache botte (éversion et rechaussement du bloc calcanéopédieux). Le contrôle radiologique a objectivé une bonne congruence articulaire après réduction (Figure 3). Une contention complémentaire par botte plâtrée a été réalisée et maintenue pendant six semaines, puis la rééducation a été entreprise. Le patient a repris son activité sportive six mois après le traumatisme. Après un recul de 12 mois, le résultat fonctionnel était satisfaisant.

## Discussion

La luxation sous-talienne est une entité rare. Hey [2], en 1803, a publié les premières observations. En 1852, Broca [3] a décrit une classification des luxations du pied qui différencie les énucléations complètes du talus des luxations sous taliennes. Baumgartner et Huguier [4], en 1907, a réalisé une étude expérimentale sur le mécanisme de luxation ce qui a permis de donner une classification anatomopathologique. C'est Allieu [5], en 1967, qui a justifié le terme de luxation astragalo-scapho-calcanéenne et en a décrit un mécanisme différent. La luxation peut être interne, externe, postérieure ou antérieure. La luxation latérale résulte d'une éversion forcée, le pied étant bloqué dans une ornière avec une pression sur la face latérale de la jambe [6]. Dans un premier temps, le ligament deltoïdien se rompt d'avant en arrière sous la poussée de la tête du talus. Ensuite, le ligament en haie se déchire par étirement, avec luxation talo-calcanéenne. Finalement, la contrainte se poursuit et la rotation interne du squelette jambier provoque la rupture du ligament talonaviculaire dorsal, permettant l'échappée totale du pied sous le talus vers le dehors. Cette luxation survient en général à la suite d'un traumatisme de haute énergie. Jard et al. [7], dans une série de 35 luxations sous taliennes, n'ont recensé que trois accidents de sport surtout le basket-ball [8]. Le diagnostic est généralement facile. Cliniquement, la déformation est majeure et le pied est fixé en éversion. La tuméfaction et l'œdème apparaissent rapidement et peuvent masquer la déformation. Le diagnostic est confirmé par une radiographie standard de profil mais surtout de face en montrant l'astragale en place dans la mortaise tibio-fibulaire alors que le pied est déjeté en dedans. La TDM permet de confirmer le diagnostic et d'apprécier les lésions ostéo-cartilagineuses associées présentes dans 20 à 30 % des cas [6, 9,10]. L'ouverture cutanée est interne dans les luxations latérales et survient dans 10 à 15 % des cas [10]. L'ischémie du pied est plus fréquente dans la luxation latérale car l'artère tibiale postérieure est prise en chevalet au niveau du talus [11].

Le traitement consiste à une réduction en urgence par la manœuvre de l'arrache botte, le genou étant en flexion pour détendre le triceps sural. L'irréductibilité doit faire suspecter une interposition et impose une réduction sanglante. 15 à 20 % des luxations latérales ne peuvent être réduites par interposition osseuse talo-naviculaire ou tendineuse (tibial postérieur, long fléchisseur de l'hallux) [10]. La stabilisation par broches, associée à une contention plâtrée de 6 semaines, réalisée par certains auteurs, est suspecte d'enraidissement mais est une garantie contre l'instabilité [11]. D'autres auteurs réalisent seulement une contention plâtrée de 6 semaines pour maintenir la réduction. La réduction doit toujours être suivie d'un contrôle radiologique pour voir l'exactitude de la réduction, rechercher les fractures associées et exclure un diastasis entre la malléole interne et le talus témoin d'une lésion du ligament deltoïdien. Le pronostic est bon si la réduction est réalisée dans les heures suivant l'accident. Les complications immédiates sont essentiellement septiques si la luxation est ouverte. L'algodystrophie est fréquente. A long terme, les patients peuvent se plaindre de douleurs et de troubles statiques. L'arthrose, très fréquente dans certaines séries (25 à 60 % des cas), touche aussi bien l'articulation subtalienne que celle de Chopart, voire l'articulation talo-crurale [6, 9,11,12]. Cependant, la raideur et l'arthrose prédominent au niveau de l'articulation subtalienne [13-15]. Le risque d'arthrose est d'autant plus grand qu'un déplacement persiste après réduction ou si le patient présente une fracture articulaire associée, plus fréquente avec la luxation latérale. La présence d'arthrose peut aussi s'expliquer par la contusion cartilagineuse, l'ischémie sous-chondrale et le dysfonctionnement mécanique par lésion du ligament en haie [12,16]. Le pied plat est la conséquence d'un défaut de cicatrisation des structures ligamentaires inférieures et postéro-médiales entraînant un affaissement de l'arche interne lors de la mise en charge. Le risque de nécrose du talus est faible car sa vascularisation est préservée dans la luxation, à l'inverse de la fracture-luxation du talus [11].

## Conclusion

La luxation sous-talienne latérale est une lésion rare et grave. Le diagnostic dépend de la qualité de l'examen radiologique pour apprécier le type de luxation et les lésions ostéo-cartilagineuses associées. Le traitement orthopédique, en urgence, consiste en une réduction suivie par une contention plâtrée pendant 6 semaines. Le traitement n'est chirurgical qu'en cas d'irréductibilité ou de fractures intra-articulaires déplacées. Le pronostic à long terme est meilleur si la luxation est interne, fermée, isolée et traitée précocement.

## Conflits d’intérêts

L' auteur ne déclare aucun conflit d'intérêts.
